# DeepStruc: towards structure solution from pair distribution function data using deep generative models†

**DOI:** 10.1039/d2dd00086e

**Published:** 2022-11-28

**Authors:** Emil T. S. Kjær, Andy S. Anker, Marcus N. Weng, Simon J. L. Billinge, Raghavendra Selvan, Kirsten M. Ø. Jensen

**Affiliations:** a Department of Chemistry and Nano-Science Center, University of Copenhagen 2100 Copenhagen Ø Denmark kirsten@chem.ku.dk; b Department of Applied Physics and Applied Mathematics Science, Columbia University New York NY 10027 USA sb2896@columbia.edu; c Condensed Matter Physics and Materials Science Department, Brookhaven National Laboratory Upton NY 11973 USA; d Department of Computer Science, University of Copenhagen 2100 Copenhagen Ø Denmark raghav@di.ku.dk; e Department of Neuroscience, University of Copenhagen 2200 Copenhagen N Denmark

## Abstract

Structure solution of nanostructured materials that have limited long-range order remains a bottleneck in materials development. We present a deep learning algorithm, DeepStruc, that can solve a simple monometallic nanoparticle structure directly from a Pair Distribution Function (PDF) obtained from total scattering data by using a conditional variational autoencoder. We first apply DeepStruc to PDFs from seven different structure types of monometallic nanoparticles, and show that structures can be solved from both simulated and experimental PDFs, including PDFs from nanoparticles that are not present in the training distribution. We also apply DeepStruc to a system of *hcp*, *fcc* and stacking faulted nanoparticles, where DeepStruc recognizes stacking faulted nanoparticles as an interpolation between *hcp* and *fcc* nanoparticles and is able to solve stacking faulted structures from PDFs. Our findings suggests that DeepStruc is a step towards a general approach for structure solution of nanomaterials.

## Abstract 2

We present a deep learning algorithm, DeepStruc, that can solve a simple nanoparticle structure directly from an experimental Pair Distribution Function (PDF) by using a conditional variational autoencoder.

## Graphical abstract

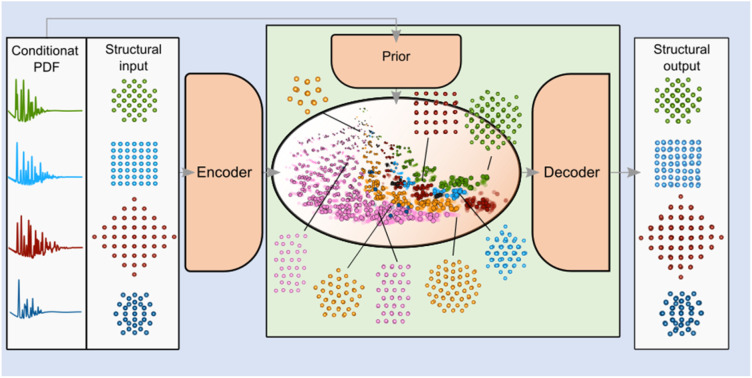

## Introduction

Crystallographic methods, such as single crystal and powder diffraction, have been foundational in the development of functional materials over the past century. They yield atomic-scale structural models for crystalline materials and allow establishing the links between material structure and properties that are at the heart of materials development.^1,2^ However, other approaches for structure determination are needed for nanostructured materials that have limited long-range order, and total scattering methods such as atomic pair distribution function (PDF) analysis have become increasingly important tools.^3–7^ Currently, PDF analysis is mainly done by fitting a known starting model to an experimental PDF, a process known as structure refinement. Recent developments in automated modelling^8–10^ have made it possible to extend the searched structural space, but identifying a model or solving a structure *de novo* from a PDF is still an enormous challenge. So far, only highly symmetrical nanostructures such as the C_60_ buckyball have been solved *ab initio* from a PDF.^11–15^ Determining the structure of less symmetrical nanostructures is limited by the lost information caused by PDF peak overlap, which challenges the use of PDF for structure solution of more complicated nanomaterials.

An approach to handle the challenges due to the information barrier in PDFs is to employ supervised machine learning (ML) methods that can learn from well-known PDF-structure pairs. In this work, we use deep generative models (DGMs). DGMs are a class of ML models that can estimate the underlying data distribution from a reasonably small set of training examples.^16^ A well-known use case of DGMs is in the generation of synthetic ‘deep-fake’ images^17,18^ based on large datasets of real images. We here train our DGM to identify new structure models by training on known chemical structures. The DGM learns the relation between PDF and atomic structure, which enables it to solve monometallic nanoparticle structures, based on PDFs it has not seen before and its learned chemical knowledge. While determining a unique structure from a PDF is not always a solvable problem, as several different structures may give rise to identical PDFs, ML methods can still learn to capture the relationship between PDF and structure and thereby push the boundaries of nanostructure solution from PDF. When there is not enough information in the PDF to provide a unique structure solution, ML methods may provide a distribution of starting models which can aid in further structure analysis.

**Fig. 1:**
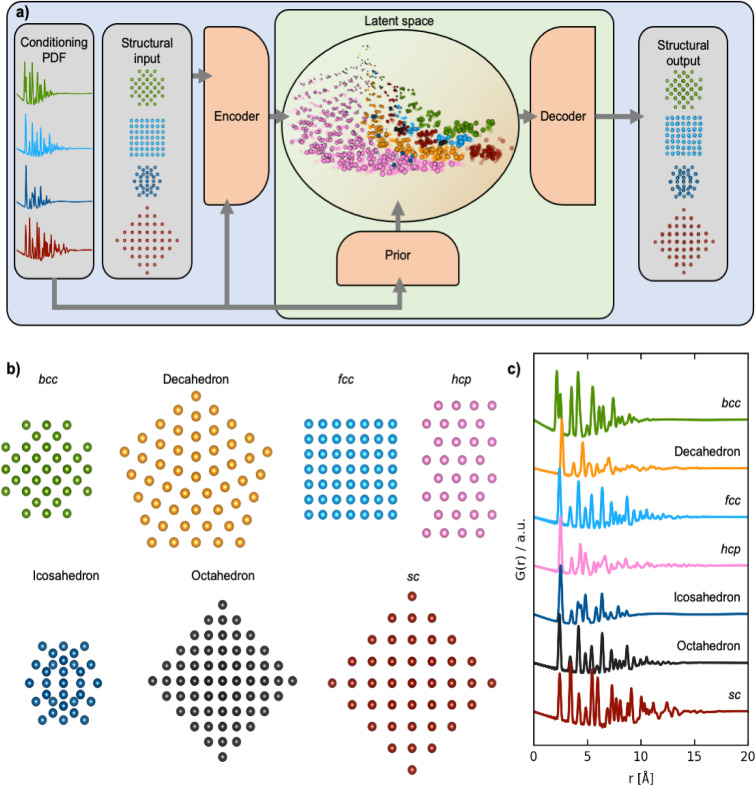
Training DeepStruc to determine the structure of MMNPs from PDFs. (a) DeepStruc predicts the *xyz*-coordinates of the MMNP structure with conditional input provided in the form of a PDF. The encoder uses the structure and its PDF as input while the prior only takes the PDF as input. To obtain the structural output a latent space embedding is given as input to the decoder which produces the corresponding MMNP *xyz*-coordinates. During training of DeepStruc both the blue and green regions are used, while only the green region is used for structure prediction during the inference process. (b) Examples of the seven different structure types which are used as input to DeepStruc together with their (c) simulated PDFs used as conditioning in DeepStruc. Each structure type has been included in the training set with varying sizes of 5 to 200 atoms and with varying lattice constants. The 3743 structures were split into training- (60%), validation- (20%), and testing sets (20%).

We apply our DGM, which we refer to as ‘DeepStruc’, for structural analysis of a model system of monometallic nanoparticles (MMNPs) with seven different structure types (Fig. 1a) and demonstrate the method for both simulated and experimental PDFs. DeepStruc is generative, which means that it can be used to construct structures that are not in the training set, *i.e.*, solve a structure from a PDF. We demonstrate this capability on a dataset of face-centered cubic (*fcc*), hexagonal closed packed (*hcp*) and stacking faulted structures, where DeepStruc can recognize the stacking faulted structures as an interpolation between *fcc* and *hcp* and construct new structural models based on a PDF.

## Methods

In the following sections, we briefly explain what a PDF is, how we obtained the simulated PDFs and their structures, and finally we elaborate on the CVAE method developed here to analyse PDFs. A more detailed description of the PDF is given elsewhere.^19^

### The pair distribution function (PDF)

The PDF is the Fourier transform of total scattering data, which can be obtained through X-ray, neutron, or electron scattering. In this work we focus on the usage of X-ray total scattering data. The scattering vector *Q* is defined as follows, where *λ* is the radiation wavelength, and *θ* is the scattering angle:
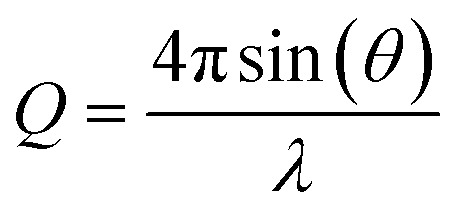


The measured scattering intensities are denoted *I*(*Q*), which are corrected for incoherent scattering, fluorescence, *etc.* and normalized such that the total scattering structure function *S*(*Q*) is obtained.
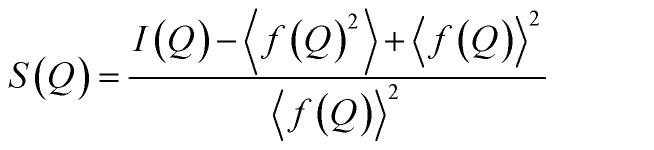
Here *f* is the atomic form factor. To obtain the structural real-space information, the total scattering structure function is Fourier transformed over the truncated *Q*-range, hence yielding the reduced PDF also known as *G*(*r*):
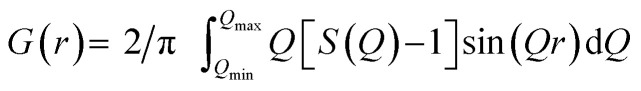


*G*(*r*) can be interpreted as a histogram of real-space interatomic distances and the information is equivalent to that of an unassigned distance matrix (uDM). All PDF simulation parameters can be found in Section G in the ESI.† The PDFs used in this project are normalised to have max(*G*(*r*)) = 1 as illustrated in Section H in the ESI.†

### Simulated and experimental data

To simulate the nanoparticles used in the training process of DeepStruc, the Python library atomic simulation environment (ASE) was used.^20^ The seven different structure types: *fcc*, *bcc*, *sc*, *hcp*, icosahedral, decahedral, and octahedral were constructed with the cluster module in ASE in the same manner as described by Banerjee *et al.*^9^ and Anker & Kjær *et al.*^21^ All MMNPs were generated in sizes ranging from 5 to 200 atoms. Each MMNP was then populated with different atoms hence changing the lattice spacing/bond distances in the MMNP. To ensure that there were no duplicate MMNPs within the dataset, all MMNPs were decomposed into a distance list of all atom–atom distances. The distance lists are a reduced format of the *xyz* representation as they are rotation- and translation-invariant in Euclidean space. All the distance-lists were sorted and duplicate structures with equivalent distance lists were removed. This yielded a total of 3742 unique MMNPs, see Section A in the ESI† for the distribution of the seven structure types. The xyz-coordinates will be the label that DeepStruc must reconstruct. Nanoparticles with each of the seven structure types can be seen in Fig. 1b along with their simulated PDF, Fig. 1a. All the simulation parameters used can been seen in Section G in the ESI.†

To further investigate the latent space behaviour of DeepStruc, a more chemically simple and intuitive dataset was made of *fcc*, *hcp*, and stacking faulted structures. *Fcc* and *hcp* can be considered layered structures that are only differentiated by the repetition of layers within the structure. *Fcc* consists of a repeated ABCABC layered structure where *hcp* is an ABABAB layered structure. A 5 layered stacking fault structure could then be described as ABCAC, as it does not satisfy either of the *fcc* or *hcp* stacking criteria. A total of 1620 stacking fault structures were generated.

### Data representation

In this work, the structures from ASE are converted into a graph-based representation in order to capture the interatomic relationships, as the original representation generated with ASE are not optimal as input to DeepStruc. Graph representations have seen increasing success in machine learning applications related to materials science as the interatomic relations in graphs are invariant to transformations of the structure such as solid translations and rotations.^22,23^ Each structure in graph representation can be described as *G* = (**X**,**A**), where **X** ∈ *ℝ*^*N*×*F*^ is the node feature matrix which contains *F* features that can describe each of the *N* atoms in the structure. We use *F* = 3 comprising only the Euclidean coordinates of the atom in a 3-dimensional space. The interatomic relationships are captured using the adjacency matrix **A** ∈ *R*^*N*×*N*^. In our case, the entries of the adjacency matrix are the Euclidean distance between each pair of atoms, resulting in a soft adjacency matrix. However, to make the adjacency matrix sparse, when the distance between any pair of nodes is larger than the lattice constant the corresponding edge weight is set to zero. When the edge weight is zero this corresponds to absence of an edge between the pair of nodes, and in other cases the edges have a weight given by the interatomic distance. Section I in the ESI† shows a decahedron consisting of seven atoms alongside the components describing it in our chosen graph representation.

### The conditional deep generative model (DGM)

DGMs such as variational autoencoders (VAEs) are commonly used to synthesize novel, synthetic data by approximating the underlying data-generating processes based on the training data.^24^ In this work, we are interested in generating structures based on properties such as the PDF resulting in the conditional DGM scenario. The specific formulation of the conditional DGM used in this work is the CVAE, initially proposed for computer vision tasks^25^ and more recently it has also been explored for synthesizing novel drug molecules.^26^ The CVAE in this work is trained to solve the unassigned distance geometry problem^27^ (uDGP) as it solves the task of converting the distances within a PDF to a chemical structure. In the uDGP the problem of taking a starting point of a list of distances and reconstructing it into a structure is broken down into two discrete problems. First, is to discover the graph that connects pairs of atoms, with the edges labelled by the distances from the distance list (the assignment problem). Second is to embed this graph into Euclidian space. An illustration of the CVAE can be seen in Fig. 1a. Here, the blue area is the training process, and the green area is the prediction/inference process. During training of the CVAE, the encoder takes pairs of structures and their corresponding PDFs as input. The encoder learns to map the structure-PDF pairs into a low-dimensional, latent Gaussian distribution, known as the encoder distribution. Each structure-PDF pair is mapped to certain regions of the latent space. When trained with large amounts of diverse data, the latent space is able to capture relationships between different structures and PDF pairs so that similar structures are closer in this latent space than very different structures. CVAEs are different from classical autoencoders in that the latent space is probabilistic, which makes it possible to sample structures from these latent encoder distributions. This is achieved during training by forcing the posterior and prior distributions to align. The prior distribution is generated with a much simpler network than that of the posterior and its only input is a PDF. The two distributions are matched by minimizing the Kullback–Leibler divergence between the encoder and prior distributions and is interpreted as the regularization term, *L*_reg_.

The prior NN gets the PDF as input and maps it to the low-dimensional prior distribution. The low-dimensional latent vector conditioned on the PDF is then input to the decoder, which is tasked to predict the *xyz*-coordinates of the structural input. During the training process, the mean squared error (MSE) between the *xyz*-coordinates of the input and output are computed to force the decoder to predict *xyx*-coordinates from the latent representations. The MSE is defined as the reconstruction loss, *L*_rec_. The CVAE is trained by jointly optimizing these two loss components:*L*_CVAE_ = *L*_rec_ + *βL*_recg_where *β* is a scaling factor that controls the relative influence of the regularization- and reconstruction-terms. In our training process, at initialization *β* is set to 0 which allows the model to focus on minimizing *L*_rec_. Each time *L*_rec_ gets below a certain threshold *β* is increased. This helps keep the model from falling into a local minimum and the process is repeated until convergence has been reached. Similar strategies for annealing *β* in VAEs have been attempted.^28,29^ At inference (test) time, the prior NN receives the PDF as input which is then mapped to the low-dimensional latent space which during training has been trained to match the encoder distribution. A sufficiently well trained CVAE is then able to predict structures from the latent space based on the PDF input. A simplified version of the CVAE used for this work, DeepStruc, can be seen in Fig. 1a. The CVAE is presented more formally in our earlier work.^21^

### Graph conditional variational autoencoder (CVAE)

In this work, two types of CVAEs were utilized depending on the type of encoder. In the conventional CVAE, the encoder was based on multi-layered perceptrons which operate on a tabular format of the node features, and the adjacency matrix populated with atom–atom distances. For the second type of CVAE – that we call the graph CVAE – the encoder consists of a graph neural network (GNN)^23,30^ and is able to process graph structured data, taking the neighbourhood information into consideration. GNNs are generalized message passing methods that can aggregate information from the neighbourhood of a node by passing messages along the edges. These messages are learned during training and can summarize the information present at the node necessary for the downstream tasks. Further, by making the encoder deep, *i.e.* adding additional GNN layers, nodes can get access to information from nodes that are farther from them. For instance, in a k-layered GNN each node had access to information from nodes that are k-hops away. In our experiments, we observed that the generative capabilities of the graph CVAE was better than the conventional CVAE, part E in the ESI.† Further, we were able to obtain comparable reconstruction quality from the graph CVAE with only two latent dimensions compared to using eight dimensions for the conventional CVAE. This indicates that the graph encoder is able to better compress the information present in the node and adjacency matrices. A minor technical detail in our CVAE models is that the predictions from the decoder do not exactly match the input features. That is, the decoder does not reconstruct the full input comprising node features and adjacency matrix but only the node features. The algorithm we refer to as DeepStruc is a graph based CVAE.

## Results and discussion

### Training DeepStruc to determine the structure of MMNPs from PDF data

DeepStruc, illustrated in Fig. 1a and discussed below, is a graph-based conditional variational autoencoder (graph CVAE). Autoencoders are a class of deep learning (DL) methods where high-dimensional inputs, such as chemical structures,^21,26^ are reduced in dimensionality. The transformation into 2 or 3 dimensional vectors is achieved using an information bottleneck by an encoder neural network (NN),^21,31,32^ and the resulting lower-dimensional, compressed feature space is known as the latent space. A decoder NN can reconstruct the input from these low-dimensional representations. When the latent space is regularized (smoothed) using normal distributions instead of discrete points we obtain a variational autoencoder (VAE). We have previously demonstrated that VAEs does a better job interpolating in the latent space compared to deterministic AEs.^21^ The VAE can be made to be dependent (conditioned) on additional information by the prior NN resulting in a CVAE.^32^

We here use MMNP structures (Fig. 1b) as input, and condition them on their simulated PDFs (Fig. 1c). The MMNP structures span seven different structure types computed using a variety of metals to emulate the variability in bond lengths in real metallic nanoparticle samples. The structure types are simple cubic (*sc*), body-centered cubic (*bcc*), face-centered cubic (*fcc*), hexagonal closed packed (*hcp*), decahedral, icosahedral, and octahedral, and all structure types have been constructed in sizes from 5 to 200 atoms. We used 3743 MMNP structures, which were randomly split into training- (60%), validation- (20%) and testing-sets (20%). Note that the validation and test sets are derived from the same underlying data distribution as the training set, and serve as intermediaries to the actual test set which is based on the experimental PDF data. A histogram of the distribution of the seven structure types are provided in Section A in the ESI.† During the training process (blue + green region Fig. 1a), DeepStruc learns to map the conditioning PDFs to their structures in the latent space. After the training process is complete, DeepStruc can be used on data that have not been part of the training set, which is referred to as ‘inference’. Further details about the DeepStruc network can be found in the Method section.

### Mapping of structures in a latent space

**Fig. 2:**
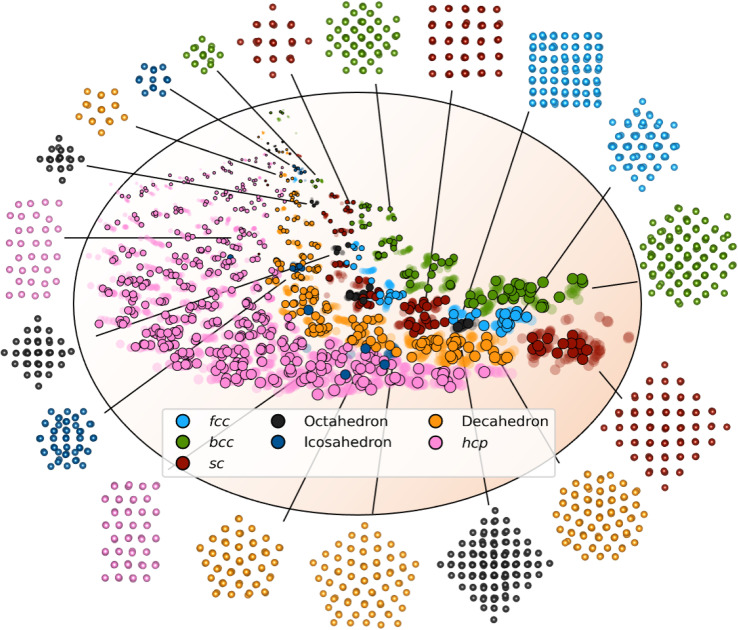
The two-dimensional latent space with structure reconstructions. The points in the latent space correspond to a structure and its simulated PDF. Data points from the test set are shown in solid colour and outlined. The points from the training and validation sets are shown as semi-transparent. The size of the points relates to the size of the embedded MMNP, and the orange background indicates the general size increase throughout the latent space. The colour of each point resemblances its structure type, *fcc* (light blue), octahedral (dark grey), decahedral (orange), *bcc* (green), icosahedral (dark blue), *hcp* (pink), and *sc* (red). Note that the structures shown here are predicted by DeepStruc during inference on PDFs from the test set.

We first evaluate DeepStruc's ability to map the MMNP structures in a low-dimensional latent space by investigating structural trends and clustering. Fig. 2 shows a visualization of the two-dimensional latent space with selected MMNP reconstructions indicated. The colour of the points indicates the structure type, and the relative point size indicates the size of the MMNP cluster. We observe that DeepStruc learns to map the chemical structures in the latent space by size and symmetry. It maps the cubic structure types (*sc*, *bcc*, and *fcc*) together, and it learns that the octahedral MMNPs are closely related to the *fcc* structure type. Interestingly, DeepStruc also allocates the decahedral structures to be in between the *fcc* and *hcp* structures. This can be rationalized by considering that decahedral structures are constructed from five tetrahedrally shaped *fcc* crystals which are separated by {111} twin boundaries that resemble stacking faults.^9,33,34^ The twin boundaries will resemble stacking faulted regions of *fcc* justifying that they exist in the latent space between *fcc* and *hcp*.

### DeepStruc for structure determination from PDF

**Fig. 3:**
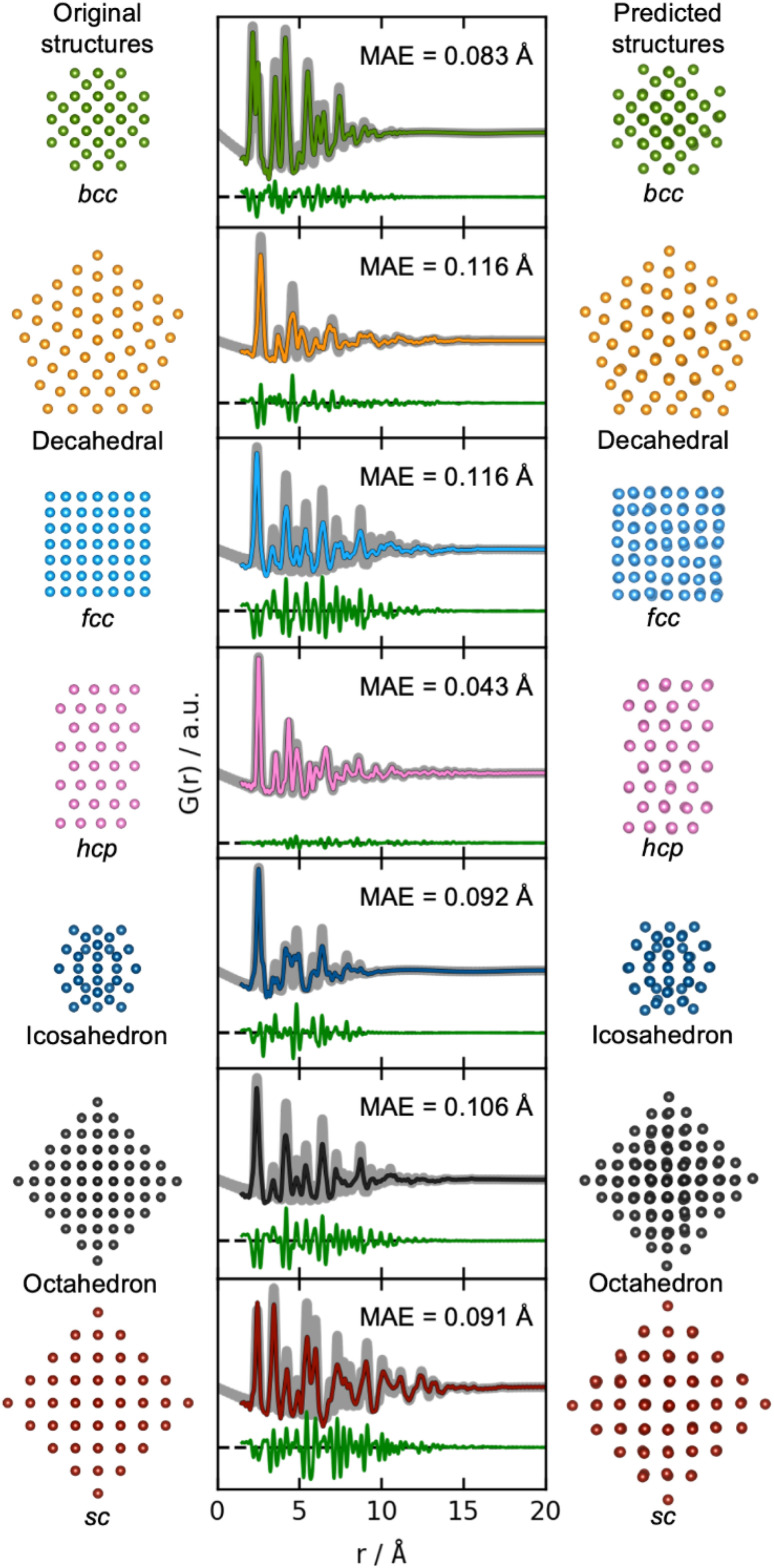
Structure determination from PDFs. Simulated PDFs (grey) from the original structures of the seven different structure types (left) are used during inference for structure prediction (right). The middle column shows the fitted PDFs of the predicted structures to the simulated PDFs of the original structures. Only the scale-factor, contraction/expansion-factor, and ADP are refined, see Section B in the ESI.†

We now move on to identify structures directly from a PDF. The results of using DeepStruc on seven simulated PDFs of MMNPs not used in the training process are illustrated in Fig. 3. Here, we show the structure that the input PDF was calculated from (left), the reconstructed structure (right), and its agreement with the input PDF after structure refinement (middle, discussed below). In all seven cases, the structures are correctly reconstructed from the PDF input. Before structure refinement, the mean absolute error (MAE) of the atom positions is 0.128 ± 0.073 Å as described in Section B in the ESI.† However, the MAE is artificially high due to a common aberration by DeepStruc, where it predicts the right geometric atomic arrangement, but isotropically contracted or expanded compared to the original structure. We do not yet understand why DeepStruc has this aberration, but it is easily solvable by refining an expansion/contraction variable as a post processing step to DeepStruc. After refining the structure to the PDF^35^ by fitting a contraction/expansion factor, a scale factor and an isotropic atomic displacement parameter (ADP), as described in Section B in the ESI,† the MAE of the atom positions is reduced to 0.093 ± 0.058 Å. The inference is thus robust against moderate changes in lattice parameter between a provided PDF and the structures that DeepStruc were trained on. The reconstructed structures exhibit some artificial positional atomic disorder that broadens the PDF peaks. The fitted ADP values (Section B in the ESI†) are thus lower than the ADP values of the conditioning PDFs.

Having established that DeepStruc works for structures highly resembling those in the training set, we now consider more challenging cases and explore the capabilities of DeepStruc on an actual test set which is far from the training distribution. As described above, the largest structures in the training set contained only 200 atoms.

**Fig. 4:**
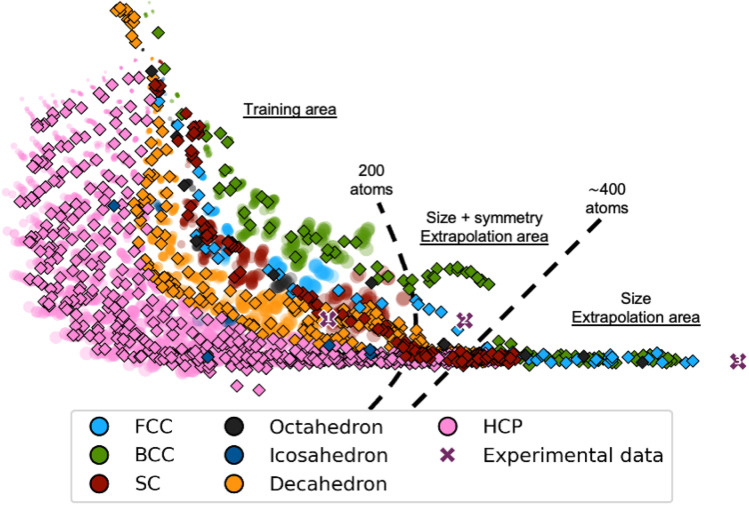
DeepStruc applied on PDFs of structures up to 1000 atoms. Each point is coloured after its structure type, *i.e. fcc* (light blue), octahedral (dark grey), decahedral (orange), *bcc* (green), icosahedral (dark blue), *hcp* (pink), and *sc* (red). Each point in the latent space corresponds to a structure based on its simulated PDF. Test PDFs from structures up to 1000 atoms are plotted as diamond markers on top of the training and validation data which are made semi-transparent. Note that the training set latent space is identical to that plotted in Fig. 2. DeepStruc has only been trained on structures up to 200 atoms. Three experimental PDFs (shown in Section C in the ESI†) obtained from differently sized *fcc* nanocrystals estimated to contain 203 (cross marker 1), 371 (cross marker 2), and 1368 (cross marker 3) atoms are illustrated as purple cross markers in the latent space.

We now evaluate it on a test set of simulated MMNPs with 5 to 1000 atoms, *i.e.*, containing much larger particles. The latent space obtained from this new test set is plotted using diamond markers in Fig. 4, where the latent space from the training process is shown with semi-transparent markers. We observe that the trends in the training area are comparable for the training set and the test set of larger MMNPs. Notably, the trends of both the size and the structure types continue beyond the training area to structures containing about 400 atoms. Beyond 400 atoms, all structure types collapse onto a line, however, DeepStruc still provides a size estimate of the structure. Of course, DeepStruc could be retrained on a larger training set if reconstructions are desired on clusters larger than 200 atoms. However, this experiment shows that DeepStruc can extrapolate significantly in the latent space. It can thereby give useful information about PDFs from structures not represented in the training set and is generative in a meaningful way. This can be compared to, for example, a tree-based ML-classifier, which is limited to a predefined structural database and cannot extrapolate. The capability of DeepStruc to extrapolate arises from each structure in the latent space being predicted as a normal distribution instead of a discrete point.

**Fig. 5:**
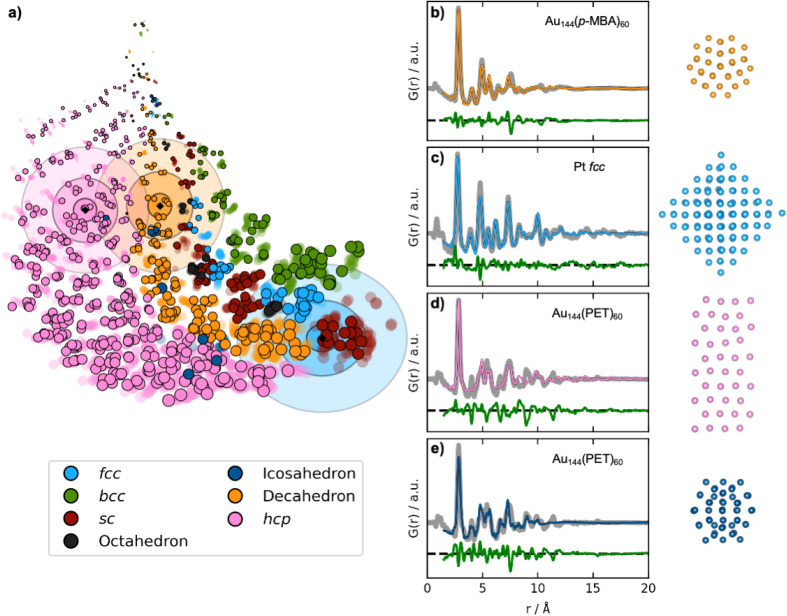
Fitting experimental PDFs with structures obtained by DeepStruc. (a) The DeepStruc latent space showing predicted latent space positions for structures from three experimental PDFs. The predicted means are shown as diamond markers, which are enclosed by three rings, indicating the sampling regions for *σ*: 3, 5, and 7. (b) PDF fit of the reconstructed structure from the Au_144_(*p*-MBA)_60_ PDF^36^ (c) PDF fit of the reconstructed structure from the 1.8 nm Pt nanoparticle PDF from Quinson *et al.*,^38^ (d) PDF fit of the reconstructed structure from the Au_144_(PET)_60_ PDF^36^ using a *hcp* structure. (e) PDF fit of the reconstructed structure from the Au_144_(PET)_60_ PDF^36^ using an icosahedral structure. Note that the test set structures shown here are the predicted structures from DeepStruc obtained during inference on experimental PDFs.

In practice, DeepStruc must be able to yield valid reconstructed structures from experimental data that contain noise and other aberrations. We therefore use DeepStruc to infer structures from previously published experimental PDFs from MMNPs. Fig. 5a shows the latent space with the predicted location of structures from three experimental PDFs. Here, the location in the latent space is represented as distributions rather than as discrete points, and multiple structures are sampled from each distribution and compared to the experimental PDF to select the best candidate. The mean of the experimental PDF distributions is represented as a black diamond with three ellipsoids indicating different confidence intervals with *σ*: 3, 5 and 7, where *σ* is the standard deviation of the normal distribution.

The first experimental dataset that we evaluate was published by Jensen *et al.*,^36^ who identified a decahedral structure as the core motif of Au_144_(*p*-MBA)_60_ nanoparticles. DeepStruc locates the Au_144_(*p*-MBA)_60_ PDF (Fig. 5b) in a decahedral region (orange distributions in Fig. 5a) in the latent space. Given the generative capabilities of DeepStruc, in theory, we can sample an unlimited number of structures for a given PDF. As described in Section D of the ESI,† we here sampled up to 1000 structures from the three normal distributions (*σ*: 3, 5, and 7), and compared their fit to the experimental PDF. Fig. 5b shows the fit of the best structural prediction, which was among the structures sampled from the *σ*: 3 distributions. DeepStruc predicts a decahedral structure, which agrees well with the literature.^36^ Other structures sampled from the three distributions are shown in Section E of the ESI,† where we also compare the DeepStruc analysis to baseline methods. We first consider a brute-force structure-mining method inspired by Banerjee *et al.*,^37^ but also compare the DeepStruc results to two simpler ML-algorithms, namely a tree-based ML classifier and a regular CVAE without a graph-based input.

The second dataset that we evaluate, published by Quinson *et al.*,^38^ are from 1.8 nm Pt nanoparticles with the *fcc* structure (described further in Section C in the ESI†). This size corresponds to *ca.* 203 atoms, *i.e.* the number of atoms in the particle goes slightly beyond the *fcc* structures in the training set that contain only 165 atoms.^38^ The location of the predicted mean is again shown as a black diamond in Fig. 5a, enclosed by three blue ellipsoids illustrating different magnitudes of standard deviation. The mean of the predicted structure is placed near the largest *sc* structures. If DeepStruc only favoured symmetry it would be placed directly on the *fcc* structures. Interestingly, DeepStruc does not purely favour size either, as it does not position the PDF near the largest structures which are *hcp* structures of 200 atoms. Instead, we observe that DeepStruc takes both symmetry and size into account by placing the mean predicted structure adjacent to the largest *sc* structures containing 185 atoms. To identify the structure from the experimental PDF, we again sample 1000 structures from the *σ*: 3, 5 and 7 distributions. When fitting these sampled structures to the dataset, we obtain the best fit from an *fcc* structure of 146 atoms that is visualized in Fig. 5c and which agrees with the baseline models (Section E in the ESI†). DeepStruc thus identifies an *fcc* structure even though the size of the MMNP is outside the training set distribution.

We also attempted to input PDFs from even larger *fcc* nanoparticles, estimated to have diameters of 2.2 and 3.4 nm, corresponding to 371 and 1368 atoms, respectively (Section C in the ESI†).^38^ Their positions in the latent space are shown in Fig. 4 along with the 1.8 nm *fcc* nanoparticles using cross markers labelled 1, 2, and 3 for increasing size. We observe that they follow the trend of the simulated *fcc* structures discussed above: while it is possible to estimate both size and symmetry for the 2.2 nm particles through extrapolation, DeepStruc can only estimate size for the 3.4 nm particle. We note that the size can be read from a PDF directly without any modelling. However, the ability of DeepStruc to predict structures on experimental data beyond those in the training set is promising for future structure solution from PDF.

While DeepStruc only has been trained on simple MMNPs, we finally evaluate it on a PDF from Au_144_(PET)_60_ nanoparticles, consisting of an icosahedral core of 54 atoms surrounded by a rhombicosidodecahedron shell of 60 atoms (Fig. 5d and e).^36,39^ We show the predicted mean position of the structure with a black diamond enclosed by pink ellipsoids. DeepStruc positions the PDF in the *hcp* region of the latent space, and when sampling 1000 structures from the distribution with *σ*: 7, the best fitting structures is an *hcp* structure with 40 atoms for the Au_144_(PET)_60_ nanoparticle (Fig. 5d). Similar structures are found when sampling from the *σ*: 3 and *σ*: 5 distributions. However, the PDF fit reveals that the reconstructed structure does not capture all peaks in the experimental PDF. When considering further the latent space, icosahedral structures are strongly underrepresented in our dataset (Section A in the ESI†) which results in an inconsistency when placing icosahedral structures in the latent space. DeepStruc is thus challenged when solving the icosahedral core structure of the nanoparticle. However, we observe that one of the test icosahedral structures is placed near the experimental PDF in latent space within the *σ*: 5 distribution. Therefore, we again try to sample 1000 structures by moving the mean of the *σ*: 3 distribution to the nearest cluster of icosahedral structures in the latent space, which are located right outside the *σ*: 7 distribution. The best fitting structure (Fig. 5e) captures all main peaks of the experimental PDF. Strategies for sampling of underrepresented structures is discussed further in Section D in the ESI.†

### Structure determination from PDF: *fcc*, *hcp*, and stacking faulted nanoparticles

To obtain a deeper understanding of the latent space's behaviour, we investigate a dataset only containing *fcc*, *hcp*, and stacking faulted structures. *Fcc* and *hcp* structures are distinguished by the stacking sequence of closed packed layers in their structures: while *fcc* structures can be described by ABCABC stacking, *hcp* structures have ABABAB stacking. Structures with other sequences are stacking faulted structures. We hypothesize that stacking faulted structures can be considered an ‘interpolation’ in the discrete space between the *fcc* and *hcp* structure type.^40^

**Fig. 6:**
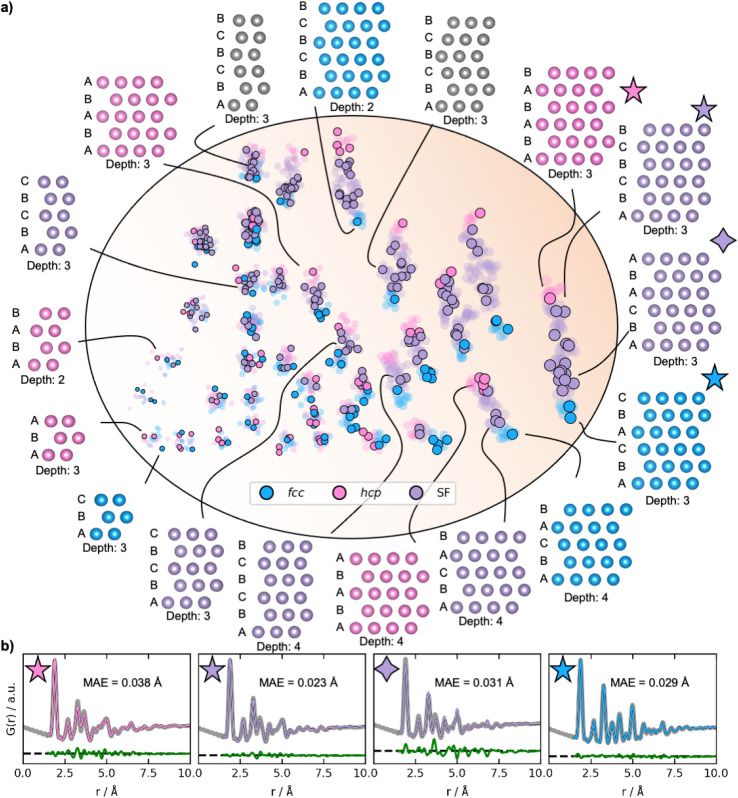
Latent space and reconstructions of stacking faulted nanoparticles. (a) The latent space and reconstructed structures shown with their stacking sequence. The structures are shown in two dimensions, and the size (number of atoms) in the third dimension is given as ‘depth’. The semi-transparent dots in the latent space represent the training and validation data, and the solid dots represent the test data. *Fcc* structures are plotted in blue, *hcp* in pink, and the stacking faulted structures in purple. The marker size represents the size of the structures. (b) Fits from reconstructed structures from the test PDF from a *fcc* (ABCABC stacking), a *hcp* (ABABAB stacking), and two stacking faulted structures. The original conditioning PDFs are shown in grey, while the PDFs of the generated structures are coloured according to their structure type. The difference curves are shown in green. The latent space is two-dimensional, hence allowing it to be directly visualized. Note that the test set structures shown here are the predicted structures obtained from DeepStruc during inference.

Examples of reconstructed *fcc* (blue), *hcp* (pink), and different stacking faulted structures (purple) and their position in the new latent space are illustrated in Fig. 6a. The MMNPs cluster in size, whilst we also observe that *fcc* and *hcp* structures separate in the latent space. It is evident that the stacking faulted structures are located in between the *fcc* and *hcp* structures in the latent space as hypothesized. It is chemically reasonable that they are positioned in this exact order based on their similarity to *fcc* and *hcp*. For example, the structure with ABCABA layers, shown in Fig. 6 with a purple star is structurally close *fcc*. We see that it is also located closer to the *fcc* structures in the latent space. On the other hand, the structure with ABCBCB layers (marked as a purple diamond in Fig. 6) can be considered structurally more closely related to *hcp* than *fcc*. DeepStruc places this structure adjacent to *hcp* structures of the same size in the latent space. DeepStruc can thus insert stacking faulted structures between *fcc* and *hcp* into the latent space in a chemically meaningful way.

Fig. 6b illustrates the fits of the reconstructed structures to the PDF data. The difference curves indicate that the predicted and true structures are very close to being identical, which is supported by the MAE of the atomic positions on 0.030 ± 0.019 Å (Section F in the ESI†). While disorder causes a broadening of the peaks, the disorder in the generated structures is minor and structures with distinct difference between the layers and in the correct sequence can be reconstructed to a satisfying degree. This is a promising result, showing that a graph-based CVAE can be used as a tool to determine the structure of stacking faulted nanoparticles from PDFs,^41,42^ which is a topic of significant current interest.^43–47^

## Conclusions

We have shown the potential of using a DGM for structure determination from simulated and experimental PDFs. Our graph-based CVAE algorithm, DeepStruc, provides valuable information through its latent space, as the MMNP structures cluster based on symmetry and size in agreement with their structural chemistry. Using experimental data, the Au_144_(*p*-MBA)_60_ nanoparticle was determined to be decahedral, Pt nanoparticles were determined to be *fcc* and the Au_144_(PET)_60_ was determined to have an icosahedral core structure, all in agreement with previous literature. While these systems are relatively simple MMNPs, we recognise that there are more complex materials where the measured PDF would not contain sufficient information to solve the structure. DeepStruc would then provide a distribution of starting models which can aid in the further structure analysis.

Our approach is only restricted by the distribution of the structural training set. When DeepStruc is trained on *fcc*, *hcp*, and stacking faulted structures, it will locate the stacking faulted structures in between the *fcc* and *hcp* structures. This suggests a strategy for training DeepStruc models on different chemical systems that also ‘interpolate’ from one to another when this can be identified. DeepStruc does not yet provide a completely general structure solution approach, but gives critical insight into how DGMs can interact with structural and diffraction information to yield candidate structures and ultimately structure solutions.

We plan to implement DeepStruc as part of PDF-in-the-cloud (PDFitc.org),^48^ where the training data can gradually be expanded over time. So far, the structures investigated are fairly ordered and contain some symmetry, but in the future, we plan to expand DeepStruc to chemical systems with more atoms and higher complexity such as metal oxide nanoparticles and alloys. Combining the PDF conditioning with data from complimentary techniques could prove important for structure determination of more complex systems. Such studies would both enable structure determination from a combined modelling perspective, but it would also reveal fundamental aspects of the information content of the different datasets for solving structure problems.

## Data availability

Code for the baseline models and DeepStruc are available at: https://github.com/EmilSkaaning/DeepStruc, https://github.com/AndySAnker/Brute-force-PDF-modelling, https://github.com/AndySAnker/MetalFinder, https://github.com/AndySAnker/CVAE.

## Author contributions

ETSK and ASA contributed to all aspects of the paper. MNW wrote the code associated to the tree-based classifier. SJLB, RS and KMØJ supervised the project. All authors contributed to the writing of the manuscript.

## Conflicts of interest

The authors declare no competing interests.

## Supplementary Material

DD-002-D2DD00086E-s001
