# Supplementary material for: DeepStruc: towards structure solution from pair distribution function data using deep generative models
Source: Digit Discov. 2022 Nov 28;2(1):69–80. doi: 10.1039/d2dd00086e (PMC9923795; doi:10.1039/d2dd00086e)
Supplement: DD-002-D2DD00086E-s001 [file DD-002-D2DD00086E-s001.pdf]

Electronic Supplementary Material (ESI) for Digital Discovery.
This journal is © The Royal Society of Chemistry 2022

Supplementary Information for:

DeepStruc: Towards structure solution from pair distribution function data using deep generative models

Emil T. S. Kjær[†1], Andy S. Anker[†1], Marcus N. Weng[1], Simon J. L. Billinge[*2,3], Raghavendra Selvan[*4,5], Kirsten M. Ø. Jensen[*1]

†Both authors contributed equally to this work.

*Correspondence to sb2896@columbia.edu, (SJLB), raghav@di.ku.dk (RS), kirsten@chem.ku.dk (KMØJ)

1: Department of Chemistry and Nano-Science Center, University of Copenhagen, 2100 Copenhagen Ø, Denmark

2: Department of Applied Physics and Applied Mathematics Science, Columbia University, New York, NY 10027, USA

3: Condensed Matter Physics and Materials Science Department, Brookhaven National Laboratory, Upton, NY 11973, USA

4: Department of Computer Science, University of Copenhagen, 2100 Copenhagen Ø, Denmark

5: Department of Neuroscience, University of Copenhagen, 2200, Copenhagen N

Table of Contents

A: Distribution of the seven structure types in data for mono-metallic nanoparticle (MMNP) structure solution 3

B: Fitting parameters and mean absolute error (MAE) measures of reconstructed MMNPs for analysis of simulated pair distribution functions (PDFs) 3

C: The Pt *fcc* Pair Distribution Functions (PDFs) from Quinson et al. 4

D: Implementation of the sampling and fitting process of the predicted structures from latent space 5

E: Comparing the DeepStruc with baseline algorithms 7

E.1.: Brute-force modelling 9

E.2.: Using a tree-based classification algorithm to predict a MMNP from a PDF 9

E.3.: Tracking the CO_2 emission of DeepStruc and the baseline models using CarbonTracker[8] 11

F: MAE measures of reconstructed stacking faulted nanoparticles 11

G: Simulation parameters of the PDFs 12

H: Normalisation of the PDFs 12

I: Graph representation of MMNPs 13

A: Distribution of the seven structure types in data for mono-metallic nanoparticle (MMNP) structure solution

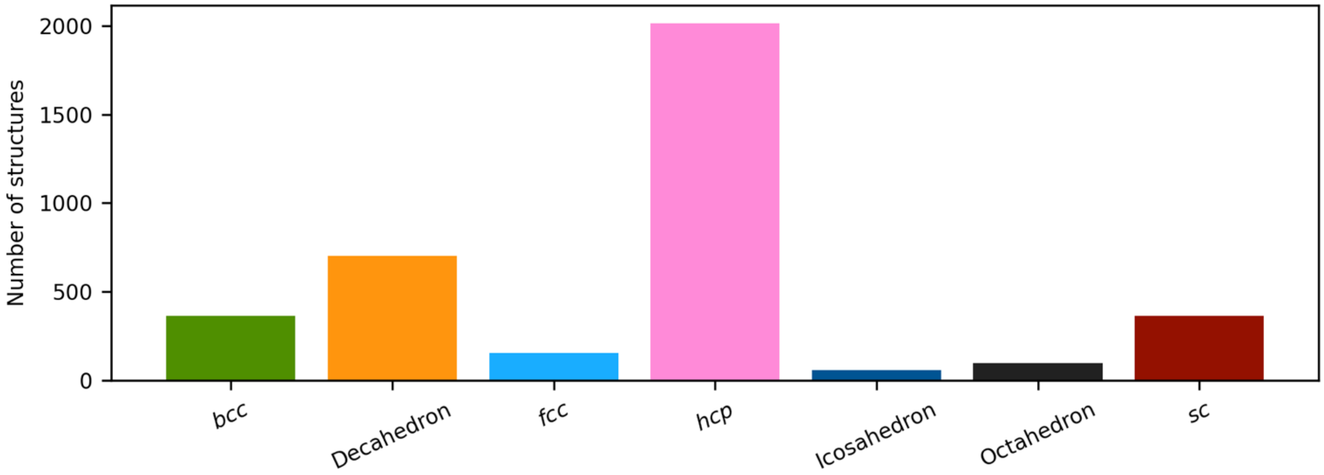

Fig. S1 | The distribution of the seven structure types in the dataset used for MMNP structure solution. In total, the dataset consists of 3742 structures, whereof 361 are body-centered cubic (*bcc*), 703 are decahedral, 152 are face-centered cubic (*fcc*), 2014 are hexagonal closed packed (*hcp*), 57 are icosahedral, 95 are octahedral and 361 are simple cubic (*sc*).

B: Fitting parameters and mean absolute error (MAE) measures of reconstructed MMNPs for analysis of simulated pair distribution functions (PDFs)

In the PDF comparisons a scale-factor, a contraction/expansion-factor and an isotropic atomic displacement parameter (ADP) are refined. This approach is taken in brute force methods such as clusterMining[1] for deciding on the best cluster for a given measured PDF. It accounts for small differences in bond-length, scale-factor, and thermal motion without changing the geometric arrangements of the atoms in the clusters

Table S1 | Refined parameters for MMNPs obtained from the DeepStruc algorithm for analysis of simulated PDFs. The MAEs for the predicted xyz-coordinates are given along with the R_{wp}-values obtained in the PDF fit. The structures are shown in Fig. 3 of the main paper.

**Name**	**#Atoms**	**Scale**	**Expansion/ contraction factor**	**B_{iso}**	**MAE [Å]**	**MAE fit [Å]**	**R_{wp} [%]**
bcc	89	0.146	0.959	0.428	0.132 ± 0.086	0.083 ± 0.068	21.2
Decahedral	105	0.107	1.030	0.238	0.140 ± 0.095	0.116 ± 0.081	39.0
fcc	171	0.117	0.957	0.284	0.182 ± 0.073	0.116 ± 0.053	54.2
hcp	128	0.082	0.974	0.081	0.093 ± 0.041	0.043 ± 0.025	10.8
Icosahedral	55	0.119	0.962	0.390	0.120 ± 0.058	0.092 ± 0.044	37.8
Octahedral	146	0.111	0.975	0.306	0.132 ± 0.056	0.106 ± 0.051	48.2
sc	177	0.158	0.984	0.210	0.091 ± 0.045	0.091 ± 0.043	46.4

C: The Pt *fcc* Pair Distribution Functions (PDFs) from Quinson et al.

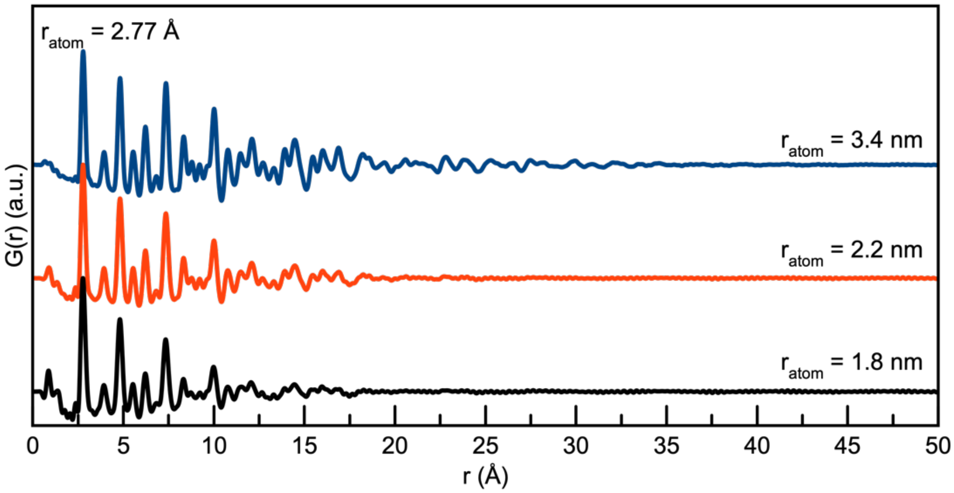

Fig. S2 | The 3 PDFs from Quinson et al..[2] The size estimate were determined by Quinson et al. by fitting the data using a spherical envelope.

The number of atoms in the particles were calculated as:

$$Atoms = \frac{V_{particle}}{V_{atom}} \cdot APF = \frac{{}^4/_3 \cdot \pi \cdot {r_{particle}}^3}{{}^4/_3 \cdot \pi \cdot {r_{atom}}^3} \cdot 0.740$$

Where V is the volume, r is the radius and APF is the atomic packing fraction which for *fcc* is 0.740. This yields a number of 203 atoms (1.8 nm), 371 atoms (2.2 nm) and 1368 atoms (3.4 nm).

D: Implementation of the sampling and fitting process of the predicted structures from latent space

During the inference process, a PDF is embedded into the latent space using a normal distribution by the prior neural network. Samples drawn from this normal distribution can be used to obtain predicted structures to be fit to the PDF. For the simulated test set (seven structure types and stacking faulted) only a single sample was drawn from the latent space to obtain structures for each of the PDFs. For the experimental data, in order to explore the latent space to a larger extent, we scale the standard deviation (σ) of the normal distribution by σ: 3, 5 and 7. We then chose to sample 1000 predicted structures for each of the three normal distributions (σ: 3, 5 and 7) and fit them to the data. For the experimental data we report the best fitted structure within each of the distributions (Supplementary Information section E). Fig. S3 demonstrates the histogram of x- and y-positions in latent space when sampling from a mean latent space position of (9.7, 14.8) using the σ: 3 distribution for the $Au_{144}(PET)_{60}$. We here sample from a normal distribution, however this could be optimized in many ways e.g. by drawing structures from a uniform distribution, by sampling from latent space regions that are well-populated by the test set, by favouring sampling from regions in the latent space that is populated by underrepresented structures, or by introducing chemical knowledge into the sampling process. Another way to alter the latent space would be to deviate from the isotropic normal distributions. The latent distribution of the CVAE is constrained to be a symmetric normal distribution (with a diagonal covariance matrix). In some cases, this might be limiting and one could consider allowing additional flexibility to the latent space structure. One of the ways of doing this is

by relaxing the diagonal covariance matrix assumption, so that asymmetric normal distributions can be modelled. This is previously studied by Jakub et al.[3] where more complex structures for the covariance matrix are modelled for VAE-type models. This additional flexibility of the latent distribution increases the complexity of the learning problem but could also provide efficient exploration of the latent space.

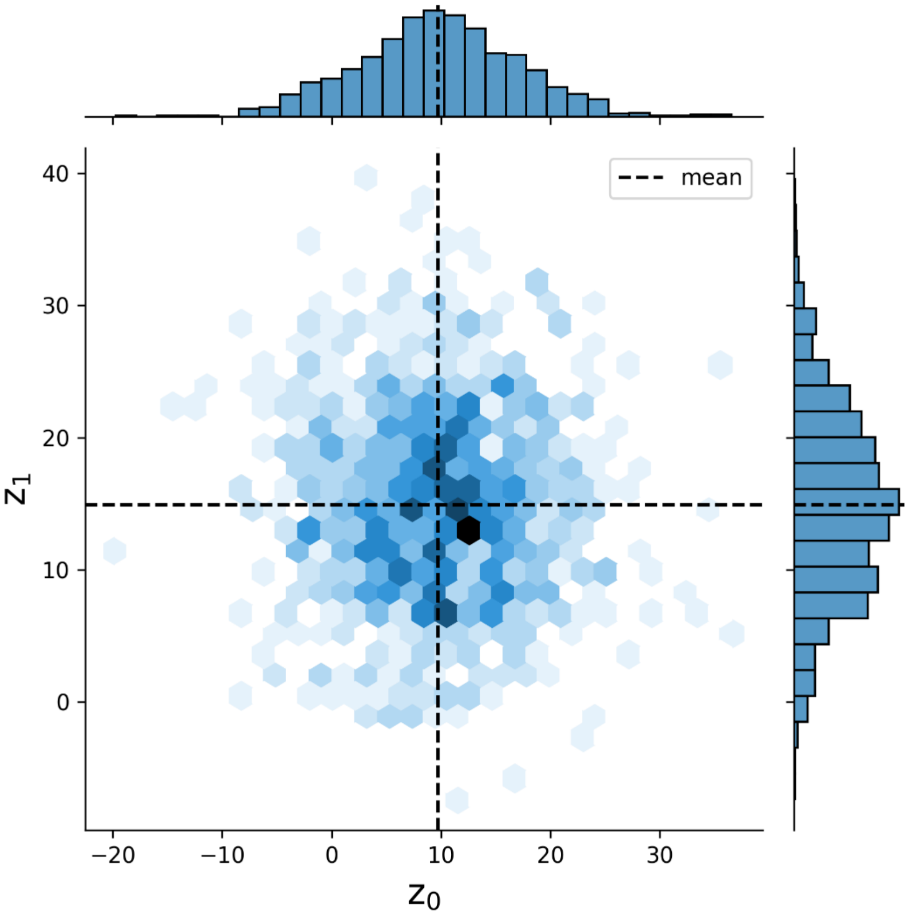

Fig. S3 | Representative histogram of z_0- and z_1-positions in latent space when sampling from a mean latent space position of (9.7, 14.8) using the σ: 3 distribution for $Au_{144}(PET)_{60}$. The black dashed line indicates the mean sampling position.

E: Comparing the DeepStruc with baseline algorithms

To evaluate the results, we compare our results with two different approaches that can be used to identify the structural model.

The first approach is the brute-force approach, further described in the part E.1, proposed by Banerjee et. al.,[1] which is fitting all constructed MMNP structures to the dataset and reporting the R_{wp} value. The second approach is a tree-based supervised learning algorithm, further described in part E.2, which has been trained on the same MMNP structures set as DeepStruc but using 100 PDFs with different simulation parameters for each MMNP structure. The range of simulation parameters used is shown in Table S5. While the brute-force method is directly providing us with the best fit, it is computationally expensive. The tree-based algorithm can, like DeepStruc, predict the chemical structure based on its PDF in less than a second. However, the brute-force approach and the tree-based algorithm cannot map a low-dimensional space of chemical structures that can be used to analyse similarities between structures. Also, they are constrained to the structural database whereas the regular CVAE without a graph-based input and the DeepStruc algorithm has generative capabilities which make it possible to both interpolate and extrapolate slightly from the training distribution. Fig. S2 illustrates a comparison of the results of the brute-force, tree-based and DeepStruc. The fits from the brute-force approach have a slightly lower R_{wp} value than the fits from the tree-based approach and DeepStruc. However, the brute-force approach is at least 3 orders of magnitude slower and consumes at least 4 orders of magnitudes more CO_2 than the Machine Learning (ML) algorithms after they have been trained, see section E.3.. The training process of the ML algorithms has to be done only once compared to the brute-force algorithm which has to be redone on every experimental dataset that is collected.

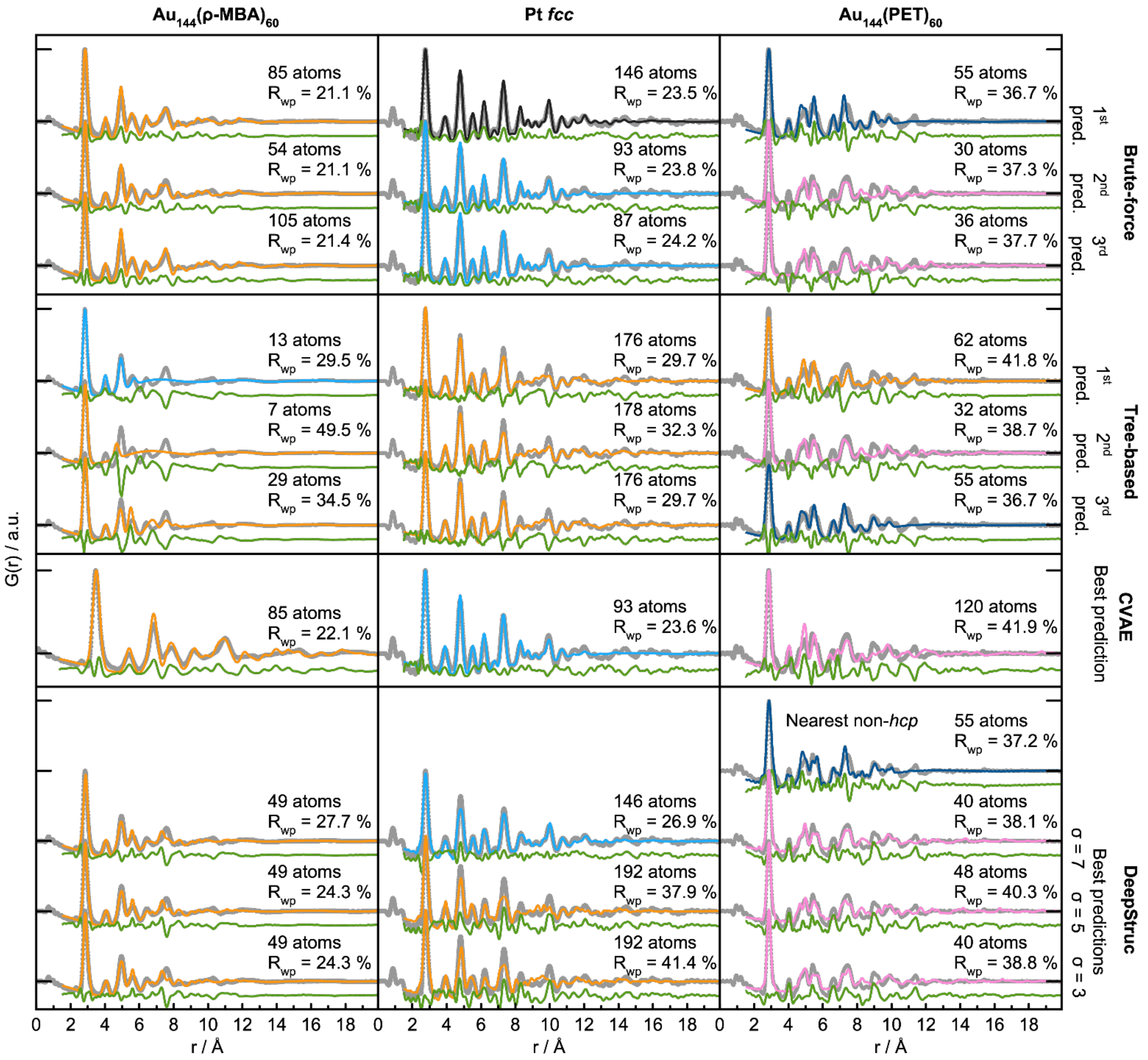

Fig. S4 | Comparing DeepStruc with baseline models. DeepStruc are used to predict a structure from each of the three different datasets, Au_{144}(p-MBA$)_{60}$, the Pt *fcc* and $Au_{144}(PET)_{60}$[2,4] as described in section D. The structures predicted by DeepStruc are compared to the top-3 predictions from the brute-force approach and the tree-based model. The fits have been made using a scaling factor, an isotropic expansion of the structure and an isotropic atomic displacement parameter (ADP) in DiffPy-CMI.[5]

E.1.: Brute-force modelling

Brute-force metal mining was done by creating MMNPs with the Python library atomic simulation environment (ASE) module[6] and fitting them to the data iteratively in DiffPy-CMI[5], from 0 to 30 Å, as proposed by Banerjee et al.[1] The advantage of the brute-force approach is that it directly yields the R_{wp} value of the fit of the structure to the dataset. The disadvantage is that it is computationally expensive. In this project, the results of the brute-force approach are used as a baseline for the predictions from the ML predictions.

E.2.: Using a tree-based classification algorithm to predict a MMNP from a PDF

A gradient boosting decision tree (GBDT) algorithm was trained to do the classification job of predicting the MMNPs from a PDF.[7] For each MMNP, 130 PDFs were simulated with parameters in the range shown in Table S5. The GBDT algorithm is trained on 100 of the PDFs, 15 of the PDFs were used for validating the model during the training process and 15 of the PDFs were used to calculate the accuracy of the model after the training process (test set). The model is trained with a learning rate on 0.15, max depth on 3 and an early stopping criteria on 5 rounds of no improvement.

Table S2 | DiffPy-CMI simulation parameters for PDF data of the data used to train the tree-based classifier.

**r_{min} (Å)**	0.0	**Q_{min} (Å^{-1})**	0–2	**B_{iso} (Å^{-2})**	0.1–1
**r_{max} (Å)**	30.0	**Q_{max} (Å^{-1})**	12–25	**Delta2 (Å^{-2})**	2.0
**r_{step} (Å)**	0.1	**Q_{damp} (Å^{-1})**	0.01–0.04		

The loss curve, Fig. S5, illustrates that the GBDT did not improve significantly after about 100 epochs.

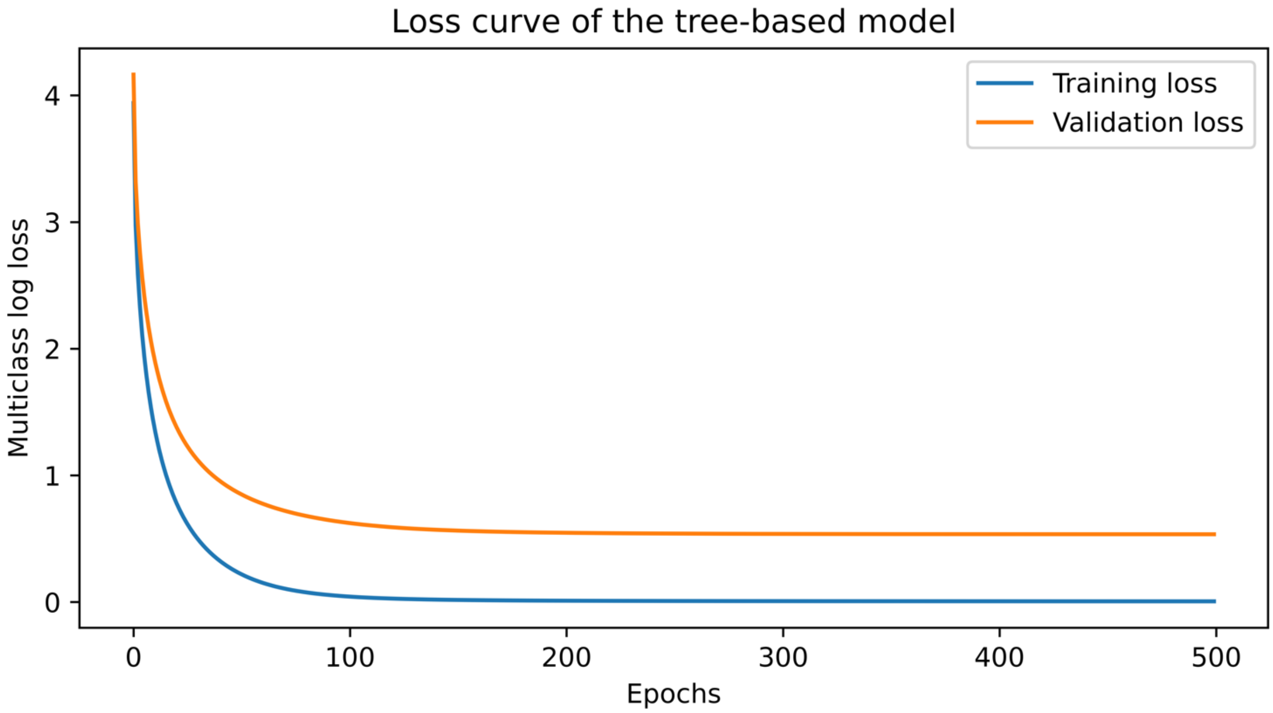

Fig. S5 | Loss curve from the training process of the GBDT.

The model subsequently predicts with an 83.87 % accuracy on the test set. Accuracy from top 3 is 95.95 % and accuracy for top 5 is 97.89 %. The GBDT algorithm does often yield very similar results to the brute-force modelling method, despite for $Au_{144}(PET)_{60}$, where it does not identify the icosahedral structure as the best fitting structure. We believe this is because the icosahedral structure was underrepresented in the model, which can be fixed with data augmentation.

E.3.: Tracking the CO_2 emission of DeepStruc and the baseline models using CarbonTracker[8]

Table S3 | CO_2 cost due to the energy consumption of the brute-force approach, the tree-based classifier and DeepStruc to determine the structure of the three experimental files - $Au_{144}(PET)_{60}$, $Au_{144}(p\text{-MBA})_{60}$ and the Pt *fcc* MMNP.[2,4] Generating the MMNPs takes about 2 hours and 53 min (0.12 kWh).

Method	Training process (kWh / min)	Prediction process (Wh / sec)
Brute-Force	-	250 / 51840
Tree-based Classifier	0.76 / 1229	0 / 0
DeepStruc	3.81 / 872	0 / 0
DeepStruc (stacking fault)	1.86 / 514	0 / 0

F: MAE measures of reconstructed stacking faulted nanoparticles

Table S4 | Refined parameters for stacking faulted structure obtained from the DeepStruc algorithm for analysis of simulated PDFs. The MAEs for predicted xyz-coordinates is given along with the R_{wp}-values obtained in the PDF fit. The structures are shown in Fig. 6 of the main paper.

Name	#Atoms	Scale	Expansion/ contraction factor	B_{iso}	MAE [Å]	MAE fit [Å]	R_{wp} [%]
HCP	72	0.007	1.010	0.193	0.047 ± 0.026	0.038 ± 0.027	15.2
SF 1	72	0.007	1.003	0.266	0.026 ± 0.016	0.023 ± 0.015	8.6
SF 2	72	0.006	1.000	0.268	0.031 ± 0.014	0.031 ± 0.014	25.6
fcc	72	0.006	1.000	0.227	0.029 ± 0.011	0.029 ± 0.011	6.8

G: Simulation parameters of the PDFs

All PDFs used for conditioning DeepStruc were simulated using the DiffPy-CMI library.[9]

Table S5 | DiffPy-CMI simulation parameters for the PDFs of the seven structure types used in Fig. 1–5.

**r_{min} (Å)**	2.0	**Q_{min} (Å^{-1})**	0.7	**B_{iso} (Å^{-2})**	0.3
**r_{max} (Å)**	30.0	**Q_{max} (Å^{-1})**	25.0	**Delta2 (Å^{-2})**	0.0
**r_{step} (Å)**	0.1	**Q_{damp} (Å^{-1})**	0.04		

Table S6 | DiffPy-CMI simulation parameters for the PDFs of the stacking faulted structures in Fig. 6.

**r_{min} (Å)**	0.0	**Q_{min} (Å^{-1})**	0.7	**B_{iso} (Å^{-2})**	0.0
**r_{max} (Å)**	20.0	**Q_{max} (Å^{-1})**	25.0	**Delta2 (Å^{-2})**	0.0
**r_{step} (Å)**	0.1	**Q_{damp} (Å^{-1})**	0.04		

H: Normalisation of the PDFs

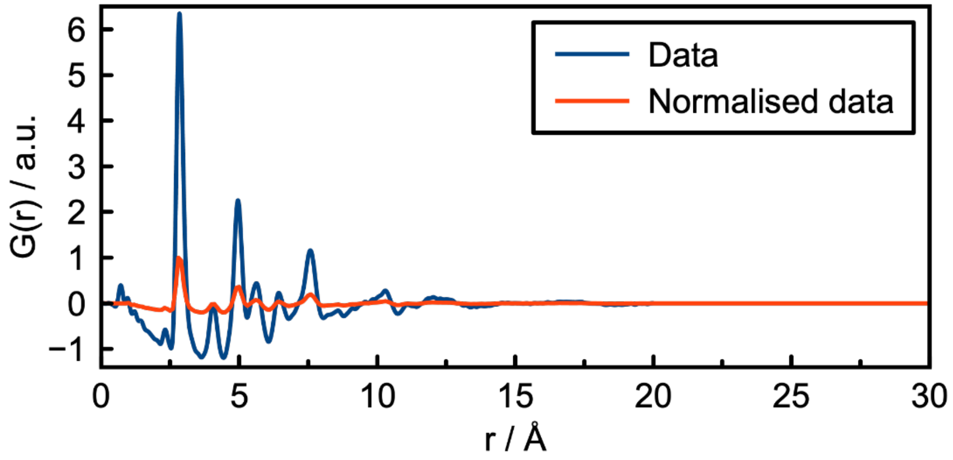

Fig. S6 | PDF and normalised PDF of the Au_{144}(*p*-MBA)$_{60}$ dataset from Jensen et al.[10]

I: Graph representation of MMNPs

Figure S7 shows a decahedron consisting of seven atoms alongside the components describing it in our chosen graph representation. Atoms 3 and 4 are connected through an edge, indicated by a yellow square in the adjacency matrix. Atoms being further separated than the lattice constant, such as atoms 3 and 5, do not have an edge indicated by the black square in the adjacency matrix. Further, the edges in our graphs are undirected as we consider them to be bonds, hence the flow of information through bonded atoms are independent of direction.

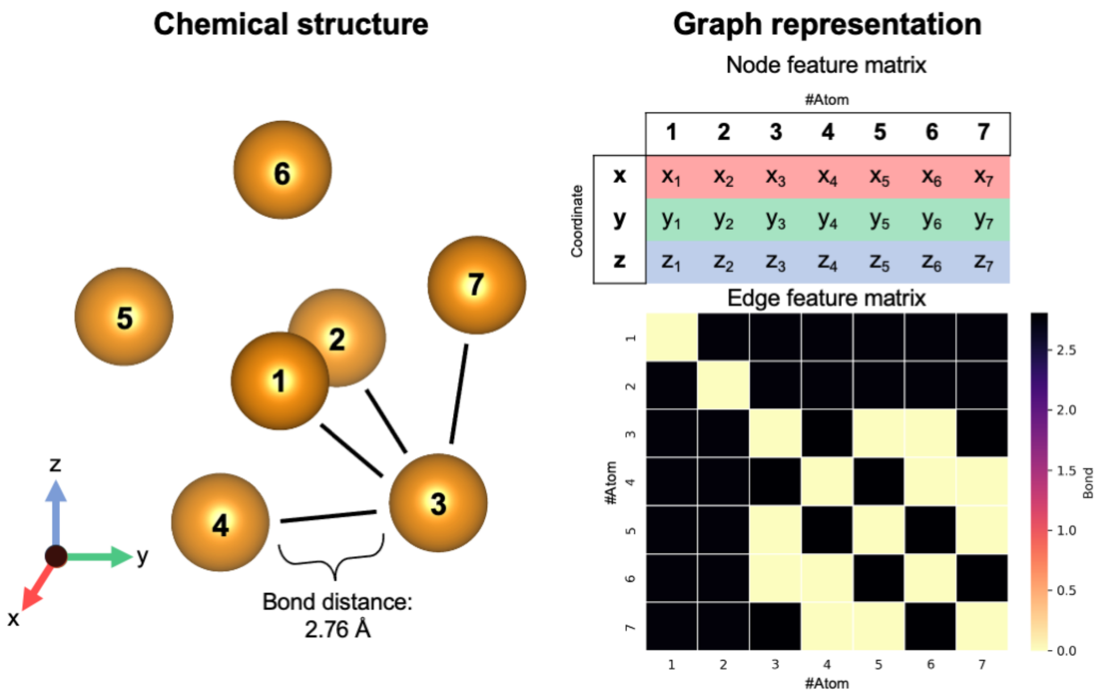

Fig. S7 | The chemical structure is shown in Euclidean space and has all atoms numbered. Atom number 3 has four neighbours each with a bond distance of approximately 2.76 Å. The graph representation is a mathematical approach to describe a chemical structure which maintains the interatomic relationship (edges) during translation, rotation and permutation. By adding the xyz-coordinates to the nodes and linking them through the adjacency matrix, the geometrical information can be transferred to the learning process of DeepStruc.

References

1 Banerjee, S., Liu, C.-H., Jensen, K. M. O., Juhas, P., Lee, J. D., Tofanelli, M., Ackerson, C. J., Murray, C. B. & Billinge, S. J. L. Cluster-mining: an approach for determining core structures of metallic nanoparticles from atomic pair distribution function data. *Acta Crystallogr. A* **76**, 24-31 (2020).

2 Quinson, J., Kacenauskaite, L., Christiansen, T. L., Vosch, T., Arenz, M. & Jensen, K. M. Ø. Spatially Localized Synthesis and Structural Characterization of Platinum Nanocrystals Obtained Using UV Light. *ACS Omega* **3**, 10351-10356 (2018).

3 Tomczak, J. M. & Welling, M. Improving variational auto-encoders using householder flow. *arXiv preprint arXiv:1611.09630* (2016).

4 Jensen, K. M. Ø., Juhas, P., Tofanelli, M. A., Heinecke, C. L., Vaughan, G., Ackerson, C. J. & Billinge, S. J. L. Polymorphism in magic-sized Au144(SR)60 clusters. *Nat. Commun.* **7** (2016).

5 Juhas, P., Farrow, C. L., Yang, X., Knox, K. R. & Billinge, S. J. L. Complex modeling: a strategy and software program for combining multiple information sources to solve ill posed structure and nanostructure inverse problems. *Acta Crystallogr. A* **71**, 562-568 (2015).

6 Hjorth Larsen, A., Jørgen Mortensen, J., Blomqvist, J., Castelli, I. E., Christensen, R., Dułak, M., Friis, J., Groves, M. N., Hammer, B., Hargus, C., Hermes, E. D., Jennings, P. C., Bjerre Jensen, P., Kermode, J., Kitchin, J. R., Leonhard Kolsbjerg, E., Kubal, J., Kaasbjerg, K., Lysgaard, S., Bergmann Maronsson, J., Maxson, T., Olsen, T., Pastewka, L., Peterson, A., Rostgaard, C., Schiøtz, J., Schütt, O., Strange, M., Thygesen, K. S., Vegge, T., Vilhelmsen, L., Walter, M., Zeng, Z. & Jacobsen, K. W. The atomic simulation environment—a Python library for working with atoms. *J. Phys.: Condens. Matter* **29**, 273002 (2017).

7 Chen, T. & Guestrin, C. in *Proceedings of the 22nd ACM SIGKDD International Conference on Knowledge Discovery and Data Mining* 785–794 (Association for Computing Machinery, San Francisco, California, USA, 2016).

8 Anthony, L. F. W., Kanding, B. & Selvan, R. Carbontracker: Tracking and predicting the carbon footprint of training deep learning models. *arXiv preprint arXiv:2007.03051* (2020).

9 Juhas, P., Farrow, C. L., Yang, X., Knox, K. R. & Billinge, S. J. L. Complex modeling: a strategy and software program for combining multiple information sources to solve ill posed structure and nanostructure inverse problems. *Acta Cryst.* **71**, 562-568 (2015).

10 Jensen, K. M. Ø., Juhas, P., Tofanelli, M. A., Heinecke, C. L., Vaughan, G., Ackerson, C. J. & Billinge, S. J. L. Polymorphism in magic-sized $Au_{144}(SR)_{60}$ clusters. *Nat Commun* **7**, 11859 (2016).
